# Clinical research evidence of cupping therapy in China: a systematic literature review

**DOI:** 10.1186/1472-6882-10-70

**Published:** 2010-11-16

**Authors:** Huijuan Cao, Mei Han, Xun Li, Shangjuan Dong, Yongmei Shang, Qian Wang, Shu Xu, Jianping Liu

**Affiliations:** 1 Center for Evidence-Based Chinese Medicine, Beijing University of Chinese Medicine, 100029, China

## Abstract

**Background:**

Though cupping therapy has been used in China for thousands of years, there has been no systematic summary of clinical research on it.

This review is to evaluate the therapeutic effect of cupping therapy using evidence-based approach based on all available clinical studies.

**Methods:**

We included all clinical studies on cupping therapy for all kinds of diseases. We searched six electronic databases, all searches ended in December 2008. We extracted data on the type of cupping and type of diseases treated.

**Results:**

550 clinical studies were identified published between 1959 and 2008, including 73 randomized controlled trials (RCTs), 22 clinical controlled trials, 373 case series, and 82 case reports. Number of RCTs obviously increased during past decades, but the quality of the RCTs was generally poor according to the risk of bias of the Cochrane standard for important outcome within each trials. The diseases in which cupping was commonly employed included pain conditions, herpes zoster, cough or asthma, etc. Wet cupping was used in majority studies, followed by retained cupping, moving cupping, medicinal cupping, etc. 38 studies used combination of two types of cupping therapies. No serious adverse effects were reported in the studies.

**Conclusions:**

According to the above results, quality and quantity of RCTs on cupping therapy appears to be improved during the past 50 years in China, and majority of studies show potential benefit on pain conditions, herpes zoster and other diseases. However, further rigorous designed trials in relevant conditions are warranted to support their use in practice.

## Background

Cupping therapy belongs to traditional Chinese medicine, the heritage from several thousand years. It is used with one of several kinds of cups, such as bamboo cups, glasses or earthen cups, placing them on the desired acupoints on patients' skin, to make the local place hyperemia or haemostasis, which can obtain the purpose of curing the diseases [1]. The earliest records of cupping is in *Bo Shu *(an ancient book written on silk), which was discovered in an ancient tomb of the *Han *Dynasty in 1973[2]. Some therapeutic cupping methods and case records of treatment were also described in early Chinese books. Zhao Xueming, a Chinese doctor practicing more than 200 years ago, completed a book named "*Ben Cao Gang Mu Shi Yi*", in which he described in detail the history and origin of different kinds of cupping and cup shapes, functions and applications [3].

There are seven major types of cupping practice in China. Usually, cupping practitioners utilize the flaming heating power to achieve suction (minus pressure) inside the cups to make them apply on the desired part of the body. This basic suction method of cupping therapy is called retained cupping, which is most commonly used in Chinese clinics as the first type of cupping. Besides this kind of suction, different types of cupping composed with different methods. The second type of cupping is bleeding cupping (or wet cupping), which contains two steps: before the suction of the cups, practitioners should make some small incisions with a triangle-edged needle or plum-blossom needle firmly tapping the acupoint for a short time to cause bleeding; the third one is moving cupping, which practitioners should control the suction by gently moving the cup toward one direction; then is empty cupping, which means the cups are removed after suction without delay; or needle cupping, which should apply the acupuncture first, then apply the cups over the needle. Cupping practitioners may also used other methods of suction, such as medicinal (herbal) cupping, which used bamboo cups, usually put the cups and herbal into a deep pan with water and boiled them together, after 30 minutes apply the cup suction on specific points according to steam instead of fire; or water cupping which is a technique involves filling a glass or bamboo cup one-third full with warm water and pursuing the cupping process in a rather quick fashion. Each kind of cupping therapy may be used for different diseases or different purposes of treatment.

Because cupping is widely used in Chinese folklore culture, the technique has been inherited by the modern Chinese practitioners. In the 1950s the clinical efficacy of cupping was confirmed by Co-Research of China and acupuncturists from the former Soviet Union, and was established as an official therapeutic practice in hospitals all over China [4]. This issue substantially stimulated the development of further cupping research.

In the context of evidence-based medicine (EBM), we need to evaluate therapeutic effect of cupping therapy to inform the practice heritage from ancient time.

## Methods

### Inclusion Criteria

Any type of clinical studies including randomized controlled trials (RCTs), clinical controlled trials (CCTs), case series (CSs), and case reports (CRs) indentifying the therapeutic effect of cupping therapy, including one or more than two types of cupping methods, compared with no treatment, placebo or conventional medication were included. Combined therapy with cupping and other interventions compared with other interventions alone were also included. Cupping therapy combined with other TCM therapies (including acupuncture) compared with non-TCM therapies were excluded. There was no limitation on language and publication type. Multiple publications reporting the same data of patients were excluded.

### Identification and selection of studies

We searched China Network Knowledge Infrastructure (CNKI) (1911-1978, 1979-2008), Chinese Scientific Journal Database VIP (1989-2008), Wan Fang Database (1985-2008), Chinese Biomedicine (CBM) (1978-2008), PubMed (1966-2008) and the Cochrane Library (Issue 4, 2008), all the searches ended at December 2008. The search terms included "cupping therapy", "bleeding cupping", "wet cupping", "dry cupping", "flash cupping", "herbal cupping", "moving cupping" or "retained cupping". Four authors (SJ Dong, YM Shang, Q Wang, and S Xu) were involved in study identifying and each of them selected one fourth of the studies for eligibility and checked against the inclusion criteria independently, they all cross checked the results with other authors.

### Data extraction and quality assessment

Four authors (SJ Dong, YM Shang, Q Wang, and S Xu) extracted the data from the included trials independently, and each of them was in charge with one fourth of the included trials. Another author (HJ Cao) checked the data and did the summary of their results. The extracted data included authors and title of study, year of publication, study design (detail of randomization if the study was RCT), type of disease, study size, age and sex of the participants, type of cupping therapy, treatment process, detail of the control interventions, outcome (for example, total effective rate), and adverse effect for each study. All data were extracted from the published studies.

Evidence from RCT is considered as gold standard for therapeutic evaluation, we specifically evaluate the methodological quality of RCT in this review. Two authors (HJ Cao and M Han) evaluated the quality of included RCTs. Assessment of methodological quality of RCTs was carried out using criteria from the Cochrane Reviewers' Handbook [5]. We assessed studies according to the risk of bias for each important outcome within included trials, including adequacy of generation of the allocation sequence, allocation concealment, blinding and outcome reporting. The quality of all the included trials was categorized to low/unclear/high risk of bias. Trials which met all criteria were categorized to low risk of bias, trials which met none of the criteria were categorized to high risk of bias, and other trials were categorized to unclear risk of bias if insufficient information acquired to make judgment.

### Data analysis and statistical methods

Data were extracted using Microsoft Access, and all the information and data were transferred into forms of Excel to be calculated for frequency. Data were summarized using risk ratio (RR) with 95% confidence intervals (CI) for binary outcomes or mean difference (MD) with 95% CI for continuous outcomes. Revman5.0.20 software was used for data analyses. Meta-analysis was used if the trials had a good homogeneity on study design, participants, interventions, control, and outcome measures. Publication bias was explored by funnel plot analysis.

## Results

### Basic information of studies

**Figure 1:**
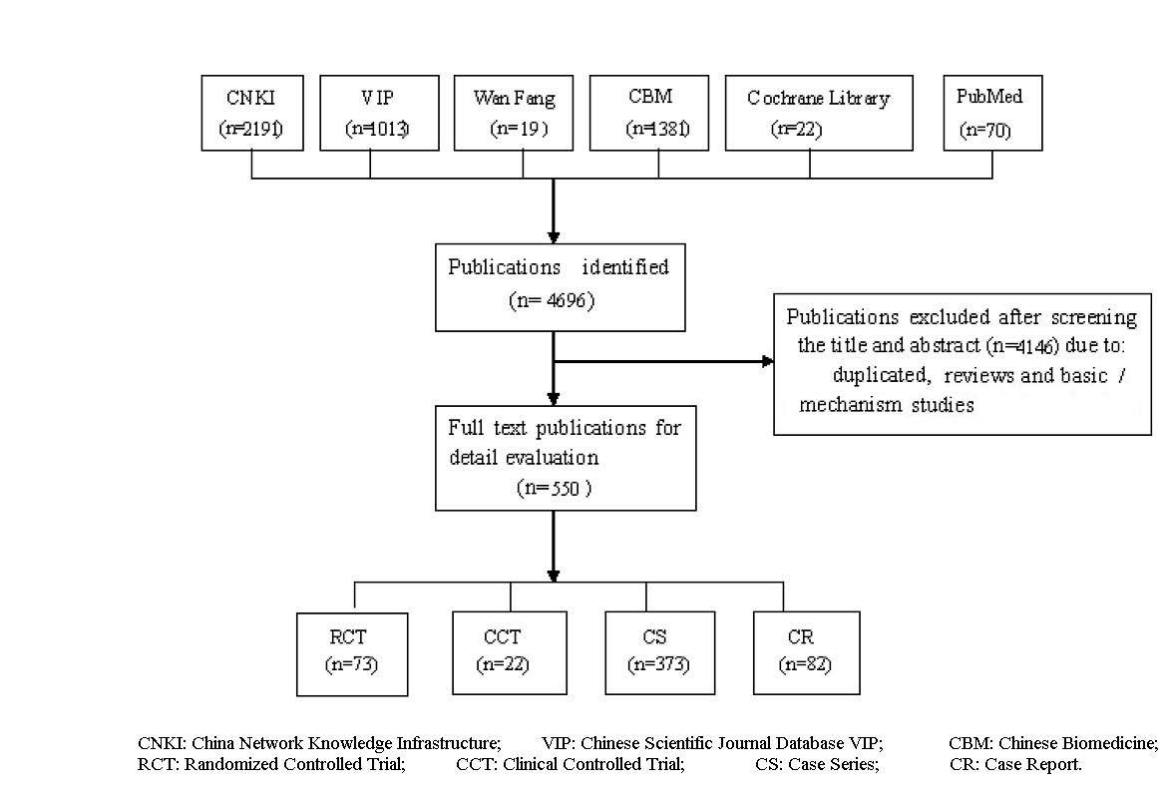
**The process of including and excluding studies**.

After primary searches from six databases, 4696 citations were identified, and the majority was excluded due to obvious ineligibility from reading title/abstract, and full text papers of 550 studies were retrieved. At last, all of the 550 studies were included in this review, which included 525 studies published in Chinese, 1 study published in English, 20 unpublished conference papers and 4 unpublished dissertation papers[7,15,30,39] (*Figure *1).

**Figure 2:**
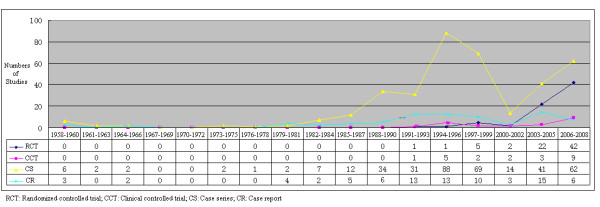
Numbers of studies on cupping therapy by study type between 1958 and 2008.

All the included studies which were published between 1959 and 2008, including 73 RCTs [6-78], 22 CCTs, 373 CSs, and 82 CRs. 214 studies (38.9%) were published between 1999 and 2008, and the number of studies has increased over the course of five decades obviously (*Figure *2). The first RCT published in 1993 and over half of the involved RCTs were reported between 2006 and 2008.

### Description of interventions

**Figure 3:**
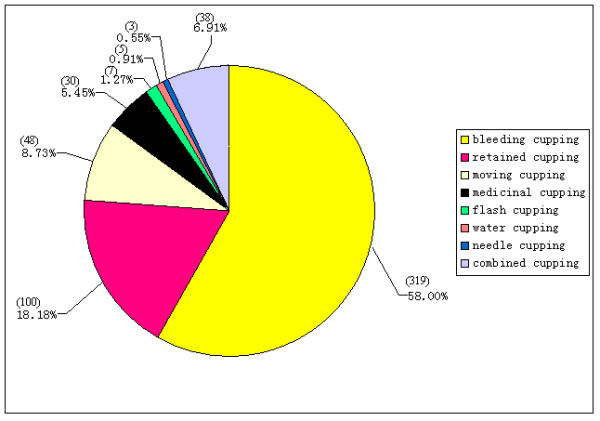
Constituent ratio of types of cupping therapy.

Among all the included studies, 319 (58.0%) used bleeding cupping as the main intervention, 100 (18.2%) used retained cupping, 48 (8.7%) used moving cupping, 30 studies (5. 5%) used medicinal cupping, 7 (1.3%) used flash cupping, 5 (0.9%) used water cupping, and 3 (0.6%) used needle cupping, combined cupping which used at least two types of cupping methods was used in 38 studies (6.9%) (*Figure *3).

### Distribution of diseases/conditions

**Figure 4:**
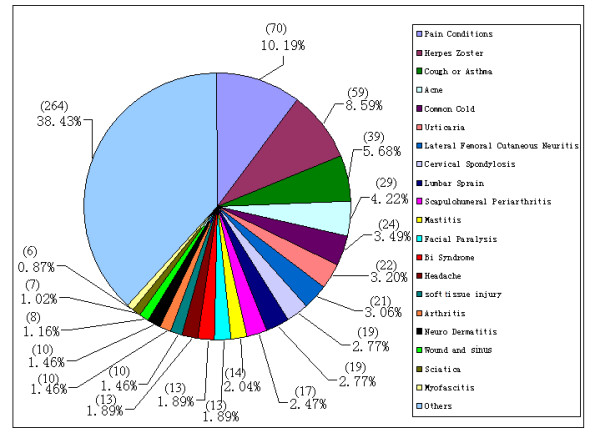
Constituent ratio of the diseases which were reported in literatures that were treated by cupping therapy.

More than 50 kinds of diseases or symptoms were treated by cupping therapy according to included studies. The top 20 diseases/conditions in which cupping is commonly employed were pain (70 studies), herpes zoster (59 studies), cough or asthma (39 studies), acne (29 studies), common cold (24 studies), urticaria (22 studies), lateral femoral cutaneous neuritis (21 studies), cervical spondylosis (19 studies), lumbar sprain (19 studies), scapulohumeral periarthritis (17 studies), mastitis (14 studies), facial paralysis (13 studies), *Bi *syndrome (Wind, cold and dampness invading the body, which is caused by changeable climate and alternate cold and heat, or dwelling in damp places, or wading, or being caught in the rain, and linger in channels and joints resulting in Bi syndrome as the result of stagnation of *qi *and blood, 13 studies), headache (13 studies), soft tissue injury (10 studies), arthritis (10 studies), neurodermatitis (10 studies), wound and sious (8 studies), sciatica (7 studies) and myofascitis (6 studies), 264 studies were concerned on other diseases treated by cupping therapy (*Figure *4).

Among the top 20 diseases in this review, 12 of them were pain related, including chronic muscle pain (100 studies, such as low back pain, skelalgia, fibromyalgia, etc); generalized pain (93 studies, such as lumbar sprain, etc); infection pain (59 studies, herpes zoster); and neuralgia pain (20 studies, such as headache and sciatica). Relieving pain was the main purpose of treating with cupping therapy of these studies. Retained cupping, moving cupping, or wet cupping therapy was usually used in these studies.

Beside pain, respiratory disease, such as common cold and symptom of cough and asthma are also treated by cupping therapy. Common cold is caused by wind and cold pathogen according to TCM theory, moving cupping along *Du *meridian may regulate the *qi*, expelling wind and clearing away cold. *Dingchuan *(EX-B1) is an acupoint belonging to Extra Meridian, which is effective on relieving asthma and cough symptoms. Retained cupping or wet cupping therapy on *Dingchuan *is usually used on cough and asthma.

Acne belongs to disorders of skin appendages, neurodermatitis and urticaria belong to disease of skin and subcutaneous tissue. All these three diseases may be caused by over heat in blood system according to TCM theory. Thereby, wet cupping therapy is popularly used for these diseases.

Facial paralysis is a kind of nerve root and plexus disorders, which belongs to disease of the nerve system. Flashing cupping and moving cupping are commonly used on this disease by regulating the circulation of *qi *and blood, expelling wind and clearing away cold, and channel meridians.

Mastitis is a kind of disease of the genitourinary system, is an inflammatory disorders of breast. Wet cupping therapy is commonly used and acupoints belonged to liver meridian are always chosen for the blood-letting before cups retained. Some of the studies also used retained cupping therapy on nipple to utilize the negative pressure to cause milk ejection, which is applied to patients with galactostasis. The same theory is used for patients with wound and sious that retained cupping therapy may help discharge of pus.

**Figure 5:**
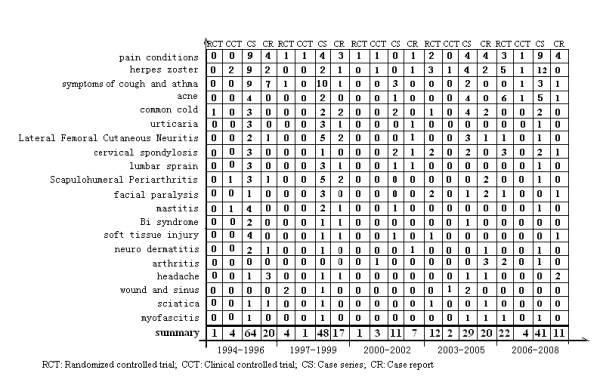
Mapping of top 20 diseases by study type between 1994 and 2008.

We also counted the number of studies of the top 20 diseases by study type between 1994 and 2008 (*Figure *5).

### Methodological quality of RCTs

According to our pre-defined methodological quality criteria, no trial could be evaluated as low risk of bias, the majority of the 73 included trials were evaluated as high risk of bias (Table 1: Reporting of five quality components in randomized clinical trials on cupping therapy). None of the trials reported sample size calculation, 15 trials described randomization procedures (such as random number table or computer generated random numbers), but none of them reported allocation concealment. Three trials mentioned blinding, but only one trial reported that they blinded outcome assessors, the other two trials did not report who were blinded. Two trials reported the number of dropouts, but none of them used intention-to-treat analysis.

**Table 1:** Reporting of five quality components in randomized clinical trials on cupping therapy

Published year	No. of Randomized controlled trials (N)	Adequate sequence generation (n/N %)	Adequate allocation concealment (n/N %)	Blinding method reported (n/N %)	Incomplete outcome data (yes) (n/N %)	Other sources of bias (yes) (n/N %)
1993-2002	6	0	0	0	0	6(100%)

2003	5	1(20%)	0	0	0	3(60%)

2004	9	0	0	0	0	3(33.33%)

2005	8	2(25%)	0	1(12.5%)	0	2(25%)

2006	16	3(18.75%)	0	1(6.25%)	1(6.25%)	2(12.5%)

2007	13	4(30.72%)	0	1(7.67%)	1(7.69%)	4(30.77%)

2008	12	5(41.67%)	0	0	0	1(8.33%)

There were 48 (65.8%) trials reported the comparability of baseline data, 18 (24.7%) trials specified the inclusion criteria, 17 (23.3%) trials specified the exclusion criteria and 48 (65.8%) trials described the diagnostic criteria. 67 (91.8%) trials reported the efficacy standard, but 51 (69.9%) out of 73 trials used composite outcome measure which categorized the effect of the treatment into four grades (cured, markedly effective, effective, ineffective) according to the change of the symptoms, the remaining 16 trials (21.9%) used single outcome measure for therapeutic effect. Symptoms were commonly used as outcome measurements, which were applied in 34 (46.6%) trials.

### Estimate effects of RCTs with cupping

Due to the insufficient RCTs and the variations in study quality, participants, intervention, control and outcome measures of the included RCTs, the results of most of the studies could not be synthesized by quantitative method. Though most of the studies showed that cupping therapy was significant effective on certain diseases, the interpretation of the positive findings from the individual studies need to be incorporated with the clinical characteristics of the included studies and evidence strength. Therefore, the conclusion of the beneficial effect of cupping therapy needs to be confirmed in large and rigorously designed RCTs.

We conducted a systematic review [80] (in press) of 8 RCTs to evaluate therapeutic effect of wet cupping therapy for herpes zoster, the meta-analyses showed that wet cupping was superior to medications for the number of cured patients (RR 2.49, 95%CI 1.91 to 3.24, *p *< 0.00001), the number of patients with improved symptom (RR 1.15, 95%CI 1.05 to 1.26, *p *= 0.003), and the incidence rate of post-herpetic neuralgia (RR 0.06, 95%CI 0.02 to 0.25, *p *= 0.0001). Combination of wet cupping and medications was significantly better than medications alone on number of cured patients (RR 1.93, 95%CI 1.23 to 3.04, *p *= 0.005), but no difference in symptom improvement (RR 1.00, 95%CI 0.92 to 1.08, *p *= 0.98).

We also conducted a systematic review [81] of RCTs to evaluate the therapeutic effect of TCM therapies for fibromyalgia, only 3 trials [82-84] on cupping therapy were included in the review according to the inclusion criteria, and two of them could be conducted in meta-analysis according to VAS (Visual Analogue Scale) and HAMD (Hamilton Depression Scale) scores after treatment. These sub-analysis of 2 out of 25 trials showed that compared to medications alone, cupping therapy combined with acupuncture plus medications was significantly better on pain relieving (MD -1.66, 95%CI -2.14 to -1.19, *p *< 0.00001) and depression remission (MD -4.92, 95%CI -6.49 to -3.34, *p *< 0.00001).

Serious adverse effects were not reported in any of the trial publications.

## Discussion

According to our findings, clinical studies on cupping therapy were obviously improved either on number or quality during the last 50 years. Though the methodological quality of the included RCTs were generally poor, some quality items showed that it was improved during the last 10 years, such as the number of the RCTs which reported the sequence generation of randomization (Table 1: Reporting of five quality components in randomized clinical trials on cupping therapy).

But we should wake up to that these studies leave much scope for well designed, conducted and reported trials. We included 550 clinical studies in this review, only 73 RCTs were published in the last two decades, 78.1% of these RCTs were with high risk of bias. According to the Consolidated Standards of Reporting Trials (CONSORT) [85], randomization methods need to be clearly described and fully reported. Although blinding of the cupping therapy might be very difficult, blinding of outcome assessors and statistics should be attempted as much as possible to minimize performance and assessment biases. Sample size calculation and analysis of outcomes based on intention-to-treat principle are important. Similar to acupuncture, cupping therapy is a kind of treatment which relevant to meridian and acupoints, so researchers may consult to the standard of STRICTA [86] on trial report, which means details of cupping treatment should be reported, such as type of cups, experience of the practitioners, period and frequency of the treatment.

About one third of the included RCTs did not report the diagnostic criteria, 63.0% of the RCTs did not report the criteria of inclusion and exclusion, and the use of composite outcome measures in 51 (69.9%) trials to evaluate overall improvement of symptoms, all the issues limit the generalization of the findings. The classification of "cure", "markedly effective", "effective" or "ineffective" is not internationally recognized, and it is hard to interpret the effect. All of the above uncertain items may increase the clinical heterogeneity. We suggest future trials completely report all the criteria they chose and comply with international standards in the evaluation of treatment effect.

We searched PubMed database using the above searching strategy, only 2 RCTs were published by international researchers outside of China until 2008. One tested wet cupping therapy on serum lipid concentrations [87], which concluded that wet cupping may be an effective method of reducing LDL cholesterol in men and consequently may have a preventive effect against atherosclerosis. Another study tested wet cupping therapy for nocturnal brachialgia paraesthetica [88], which suggested short-term effects of a single wet cupping therapy. Meanwhile, two further RCTs with cupping originating outside China have been published after 2008, demonstrating increasing interest in this field. One trial [89] found that traditional wet-cupping care was significantly more effective in reducing bodily pain than usual care at 3-month follow-up with satisfactory safety and acceptance to patients with nonspecific low back pain. Another trial [90] investigated the effectiveness of cupping therapy with the conclusion that cupping therapy may be effective in relieving pain and other symptoms related to carpal tunnel syndrome (CTS), however, the efficacy of cupping in the long-term management of CTS and related mechanisms remains to be clarified. We are glad to see that these trials are apparently with good methodological quality, however, though most of the clinical trials showed positive results on therapeutic effect of cupping therapy, the appropriate duration of the cupping therapy, the syndrome differentiation for acupoints selection, and the frequency of the cupping therapy were unclear according to current evidence. Future studies should address these issues.

This review suggests that there is insufficient high-quality evidence to support the use of cupping therapy on relevant diseases. Although quite a number of clinical studies reported that cupping therapy may have effect on pain conditions, herpes zoster, symptoms of cough and asthma, acne, common cold, or other common diseases. The current evidence is not sufficient to allow recommendation for clinical use of cupping therapy for the treatment of above diseases of any etiology in people of any age group. The long-term effect of cupping therapy is not known, but use of cupping is generally safe based on long term clinical use and reports from the reviewed clinical studies.

The number of RCTs on treatment using cupping therapy is scarce in terms of a specific disease. Existing trials are of small size and low methodological quality. Further high quality studies of larger sample size are needed to assess the effectiveness of cupping therapy. It might be worthwhile to examine the effectiveness of cupping therapy or combination of cupping therapy with other non-pharmacological or pharmacological treatments for pain conditions, herpes zoster, symptoms of cough and asthma, acne, common cold, or other common diseases which were most treated by cupping therapy according to this review. In addition, the methodological quality should be improved, and the study design and report should also be standardized. The protocol of the study should be registered in authoritative organizations [91], such as WHO International Clinical Trial Registration Platform (WHO ICTRP).

## Competing interests

The authors declare that they have no competing interests.

## Authors' contributions

HC participated in the design of the study, searched studies, participated in extracted data, assessed study quality, analyzed data, performed the statistical analysis and drafted the manuscript. MH participated in extracted data, assessed study quality. XL co-developed the full text of the review. SD, YS, QW, SX participated in searched literature, identified clinical studies for inclusion and extracted data. JL conceived of the study, and participated in its design and coordination, co-developed the full text of the review and is the corresponding author.

## Pre-publication history

The pre-publication history for this paper can be accessed here:

http://www.biomedcentral.com/1472-6882/10/70/prepub
